# Evolutionary conserved compositional structures hidden in genomes of the foot-and-mouth disease virus and of the human rhinovirus

**DOI:** 10.1038/s41598-019-53013-8

**Published:** 2019-11-12

**Authors:** Miguel Angel Fuertes, Silvia López-Arguello, Carlos Alonso

**Affiliations:** Centro de Biología Molecular “Severo Ochoa” (CSIC-UAM), Universidad Autónoma de Madrid, c/Nicolás Cabrera 1, 28049 Madrid, Spain

**Keywords:** Evolutionary biology, Molecular evolution

## Abstract

*Picornaviridae* family includes several viruses of great economic and medical importance. Among all members of the family we focused our attention on the human rhinovirus, the most important etiologic agent of the common cold and on the foot-and-mouth disease virus that cause of an economically important disease in cattle. Despite the low sequence similarity of the polyprotein coding open reading frames of these highly divergent picornaviruses, they have in common structural and functional similarities including a similar genomic organization, a capsid structure composed of 60 copies of four different proteins, or 3D-structures showing similar general topology, among others. We hypothesized that such similarities could be reflected in emergent common compositional structures interspersed in their genomes which were not observed heretofore. Using a methodology categorizing nucleotide triplets by their gross-composition we have found two human rhinoviruses sharing compositional structures interspersed along their genomic RNA with three foot-and-mouth disease viruses. The shared compositional structures are in one case composed by nucleotide triplets containing all nearest-neighbours of A and G and in other case containing all nearest-neighbours of A, and C. The structures are under strong evolutionary constraints for variability, allowing the access to novel viral genomic motifs with likely biological relevance. The conserved fragments would be useful to predict critical mutation points sites important from the evolutionary point of view.

## Introduction

*Picornaviridae* is a family of small, viruses that are important disease-causing agents in human and animals^1^. Its significance in economy and medicine has contributed considerably to the progress of virology^2^. Picornaviruses share a similar icosahedral capsid containing a small RNA genome with the VPg protein covalently attached to its 5′-terminus and a 3′-terminal poly(A) tail.

In this paper, we centre the attention in the study of four serotypes of the human rhinovirus (HRV) and four of the foot-and-mouth disease virus (FMDV). High-resolution structures of some HRV and FMDV serotypes have been solved by x-ray diffraction^3,4^. HRV and FMDV capsids have 60 protomers composed of four different proteins (VP1-VP4). The capsid is organized during reassembly on a pseudo T = 3 symmetry (P = 3) with a diameter of 27–30 nm, associating five protomers to form a pentameric structure and 12 pentameric structures to form the capsid. HRV and FMDV require of the proteolytic cleavage of the capsid polyprotein P1 to obtain the protein subunits. The coat proteins VP1, VP2, and VP3 have a similar characteristic fold and VP4 is an internal protein. HRV and FMDV have evolved in different ways to penetrate host cells recognizing a variety of receptors: HRV uses an intercellular adhesion protein^5^ and FMDV predominantly uses integrins^1,6^.

Viruses from the *Picornaviridae* family display high genetic variability and phenotypic flexibility, sometimes complicating their identification and classification^7,8^. Changes in some phenotypic features do not correlate faithfully with speciation supporting the idea that those phenotypic features respond rapidly to selection and consequently, viruses from the *Picornaviridae* family could exploit many adaptive solutions independently of their evolutionary history. The discordance in tree topology among datasets, underline differences in evolutionary parameters as selection pressure, rates of evolution, and the possibility of recombination events in the past^9^. Particularly, the apparent lack of similarity among genomes and the fact that some homologous sequences not always reflect significant sequence similarity^9^ encourage to suggest, as hypothesis, that some common compositional structure not observed previously at RNA level could be shared by those genomes along their evolutionary history. The polyprotein coding ORFs of FMDV and HRV were analyzed by the triplet-composon method^10,11^ by reading the RNA sequence in a fully overlapping way to avoid information loss and guaranteeing all triplets of the sequence are considered in the study^11^. The triplets are organized in 14 categorizations, called triplets composons (tCPs) containing useful evolutionary information^11–13^.

Taking into account all these considerations, the objective of the paper has been to determine whether such emergent compositional structures exist in picornaviruses (HRV and FMDV) and if so, to study its composition and distribution along the open reading frame (ORF) encoding their polyproteins. FMDV and HRV were chosen as model picornaviruses because of their economical importance^14,15^ and the vast amount of genetic and functional knowledge available on these viruses.

## Methods

### Polyprotein coding ORFs, information and controls

The serotypes analysed of the *Picornaviridae* family were: HRV serotype C, strain QCE (HRV-C), locus GQ323774; HRV14 serotype B (from the major receptor group (HRV14), locus K02121; Human rhinovirus A, strain HRV_A_SK001 (HRV-A), locus MH899591; HRV2 serotype B (from the minor receptor group (HRV2), locus X02316; FMDV serotype A, strain KEN/K74/2016 (FMDV-A), locus MN116688; FMDV virus serotype C, isolate C-S8, clone 1 (FMDV-C), locus AJ133357; FMDV serotype SAT 1 isolate SAT1/NIG/4/15 (FMDV-SAT1), locus MF678826; and FMDV serotype O isolate O1Campos (FMDV-O), locus AJ320488. This selection was based on the convenience of including a large enough number of serotypes of each virus: four HRV serotypes included in three evolutionary subfamilies (A, B and C) HRV-A, HRV2 minor receptor group, HRV14 included in HRV-B and HRV-C and four serotypes included in the most important evolutionary groups of FMDV as FMDV-A, FMDV-C, FMDV-O and FMDV-SAT1^14,16^.

The sequence of picornaviruses polyprotein-coding ORFs have been obtained from the GenBank® https://www.ncbi.nlm.nih.gov/^17^. Abbreviations are taken from the International Committee on Taxonomy of Viruses (ICTV) https://talk.ictvonline.org/^18^. In the paper, T is listed as a nucleotide. Since the viral genomes are RNA, U should be substituted for T. However, very often, RNA is reverse-transcribed into DNA first, and then the DNA is sequenced. This is the main reason why GeneBank present the single-stranded RNA viral genomes in this way. Moreover, NCBI support indicates that replacing U with T is a GenBank convention, which saves computational resources.

To discard the possibility of random fits, we compared the average of 5 randomized sequences generated by the tCP-sequence of the serotypes of HRV and FMDV polyproteins coding ORFs with their respective tCP-sequences, using the application *shuffleseq* from the European Molecular Biology Open Software Suite (EMBOSS)^19^ located in http://emboss.bioinformatics.nl/ that shuffle a set of sequences maintaining composition. Averaging of the 5 randomized genomes, we would blunt any spurious signal but potentiate any underlying signal present on the individual randomized genomes, if any. Finally, two identical polyprotein coding ORFs were compared to test the reliability of the software.

### Numerical analysis

Similarities and dissimilarities among polyprotein coding ORFs of these serotypes of HRV and FMDV were analyzed by the tCP-method^10,11^. The justification of the method is based in the existence of exclusionary multiplet categorizations characterized by the presence or absence of particular bases. There are 14 different categorizations with a multiplet length of 3 NTs (a triplet)^10^. The categorizations found were called triplet-composons (tCPs). In this method the RNA sequence is read in a fully overlapping way to avoid information loss, guaranteeing that all triplets of the sequence are considered in the study. This way of reading takes into account the context of each nucleotide in the RNA sequence^11^. Abbreviations and sets of triplets associated to each tCP are indicated in Table 1.

**Table 1:** List of all tCPs and their associated NT-triplets (adapted from^10,36^).

tCPs	NT-triplets per tCP
**Non degenerated**	
<A>	AAA
<T>	TTT
<G>	GGG
<C>	CCC
**Degenerated**	
<AC>	AAC, CAA, ACA, CCA, ACC, CAC
<AT>	AAT, TAA, ATA, TTA, ATT, TAT
<AG>	AAG, GAA, AGA, GGA, AGG, GAG
<CG>	CCG, GCC, CGC, GGC, CGG, GCG
<GT>	GGT, TGG, GTG, TTG, GTT, TGT
<CT>	CCT, TCC, CTC, TTC, CTT, TCT
<AGC>	AGC, GCA, CAG, ACG, CGA, GAC
<AGT>	AGT, GTA, TAG, ATG, TGA, GAT
<ACT>	ACT, CTA, TAC, ATC, TCA, CAT
<TCG>	TCG, CGT, GTC, TGC, GCT, CTG

**Figure 1:**
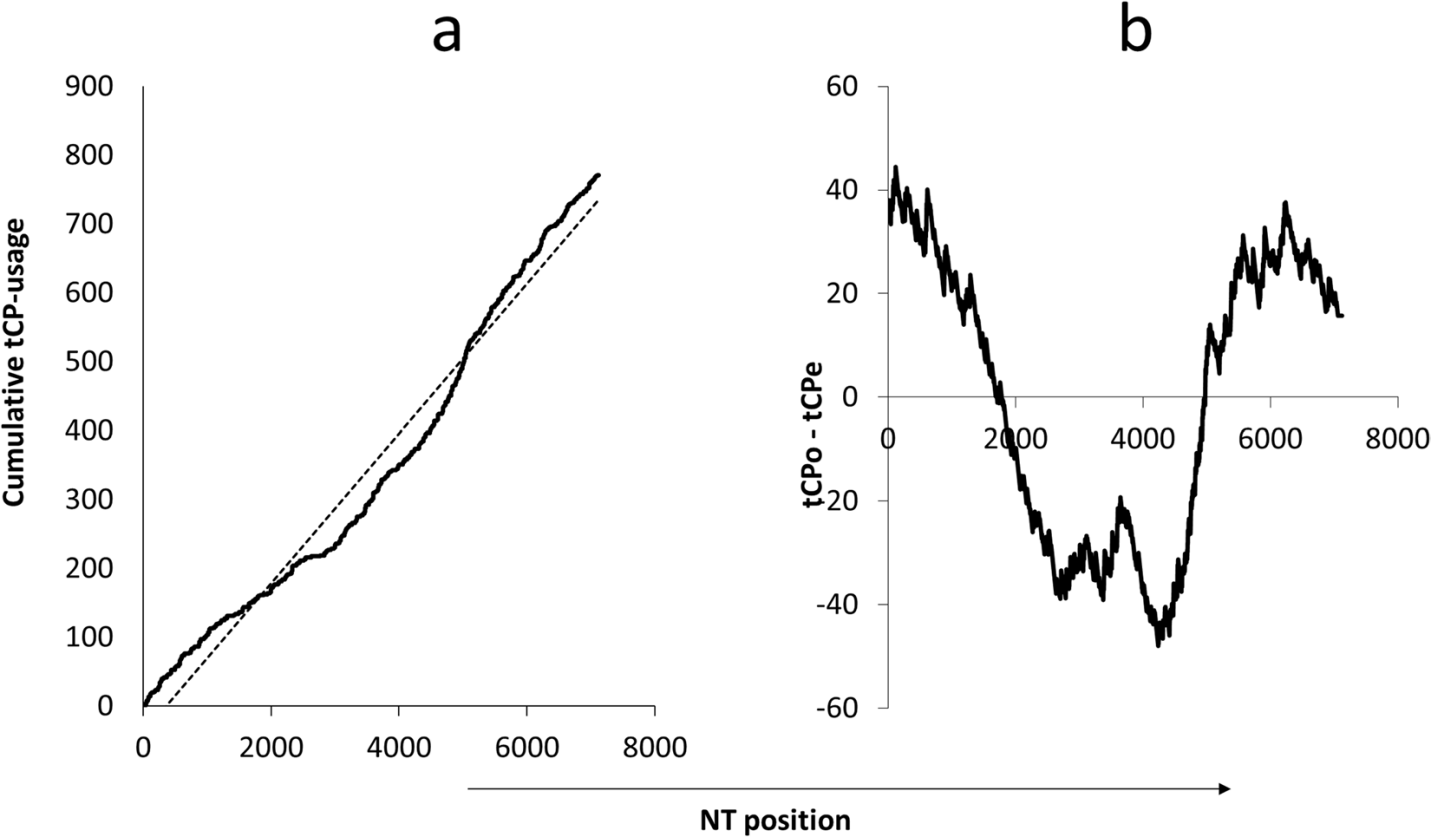
Cumulative tCP-usage frequency graphs with FMDV-polyprotein length. (**a**) Cumulative <AG>-usage frequency graph with FMDV-polyprotein length and the corresponding regression line (----). (**b**) Projection of the regression line and cumulative data on the length axis (here after named *tCP-profile*).

To study the distribution of tCPs along the genomes of the serotypes of HRV and FMDV polyprotein coding ORFs, we will use a variant of the original method^11^ consisting in the analysis of the tCP-distribution along the polyprotein coding ORF by registering (Fig. 1a) the cumulative tCP-usage frequency^13^. The cumulative frequency is the sum of all previous tCP-appearances along the sequence up to the current length. To facilitate the study of the distribution of tCPs along the polyprotein coding ORFs (Fig. 1b) the cumulative tCP-graph and its regression line (Fig. 1a) were projected on the length axis^13^ by subtracting the cumulative tCP-usage frequency from the regression line (termed *tCP-profile*). The tCP-profile represents the distribution of differences between the tCP-events observed, tCPo, and the tCP-events estimated, tCPe. We will consider that the two RNA sequences to be compared share a similar tCP-profile when their Pearson correlation coefficient (r) is equal to or higher than an arbitrarily cut-off (in this case r ≥ 0.85). This cut-off has been chosen because it is high enough to eliminate spurious fits^13^. Graphs and statistics were carried out with the package OriginPro 8 SRO V8.0724 (B724) ©OriginLab Corporation.

### Distribution of tCPs along the sequence of the polyprotein-coding ORF

The distribution of tCPs along the polyprotein coding ORFs was determined as follow: (i) using Table 1, we translated the HRV and FMDV polyprotein coding ORFs to tCP-sequences; (ii) we compared the tCP-sequences with the dynamic algorithm created by Needleman-Wunsch for the global alignment of two sequences^20,21^; (iii) to compare the tCP-profiles of the serotypes of HRV and FMDV we used graphical representations computing the correlation coefficient between them; (iv) Then, we located along the polyprotein-coding ORFs any shared RNA fragments corresponding to the conserved tCPs in those cases where the correlation coefficient was higher than the cut-off. The threshold for significance in this paper was a P-value of 0.01.

## Results

### Comparison of the NT- and tCP-sequences of HRV and FMDV polyprotein coding ORFs

Table 2 shows the percentages of identity and gaps, and the number of shared tCPs of HRV and FMDV polyprotein coding ORFs. The comparison of the eight divergent serotypes of FMDV and HRV considered in the study revealed that the four serotypes of FMDV show higher similarity than the four serotypes of HRV, indicating that between the serotypes of FMDV there is lower divergence than between serotypes of HRV^22,23^. Low degree of similarity, like that obtained at random, was observed when the serotypes of HRV and FMDV RNAs were compared. Table 2 also shows the existence of lower identity and higher percentage of gaps in the tCP-sequence alignments, than in the NT-sequence alignments. It was previously reported that the decrease observed in tCP-identity relative to that observed in NT-sequences is a consequence of the fully overlapping reading of tCP-sequences^11^. As expected, the highest similarity corresponds to identical sequences (100% identity, 0% gaps) as observed, for example, in HRV14. The same occurs for the remaining serotypes analysed.

**Table 2:** tCPs shared and percentages of identity and gaps between the serotypes of HRV and FMDV polyproteins encoding ORFs. Comparison with their random (RND) generated polyproteins encoding ORFs.

Organisms	NT-identity (%)	NT-gaps (%)	tCP-identity (%)	tCP-gaps (%)	tCP shared
HRV14 and HRV14	100	0	100	0	14
HRV14 and HRV-C	57	24	33	31	4
HRV14 and HRV-A	56	26	33	32	1
HRV14 and HRV2	57	24	33	31	1
HRV-C and HRV-A	57	23	33	32	3
HRV-C and HRV2	56	25	35	33	1
HRV-A and HRV2	75	4	33	32	2
FMDV-O and FMDV-A	84	3	66	8	6
FMDV-O and FMDV-C	85	3	70	5	6
FMDV-O and FMDV-SAT1	75	8	53	15	3
FMDV-SAT1and FMDV-C	75	8	51	19	5
FMDV-SAT1and FMDV-A	75	7	53	16	4
FMDV-C and FMDV-A	84	3	35	9	5
FMDV-C and HRV14	43	44	25	44	1
FMDV-A and HRV14	43	43	25	44	1
FMDV-A and HRV-C	43	44	26	44	0
FMDV-A and HRV-A	42	45	25	42	0
FMDV-A and HRV2	43	44	26	44	0
FMDV-C and HRV-C	43	44	25	45	1
FMDV-C and HRV-A	42	45	24	44	0
FMDV-C and HRV2	43	43	25	45	0
FMDV-O and HRV14	42	44	25	44	0
FMDV-O and HRV-C	42	46	26	44	0
FMDV-O and HRV-A	42	44	24	47	0
FMDV-O and HRV2	43	43	26	44	0
FMDV-A and RND FMDV-A	43	45	23	40	0
FMDV-C and RND FMDV-C	42	46	26	47	0
FMDV-SAT1 and RND FMDV-SAT1	41	44	24	40	0
FMDV-O and RND FMDV-O	42	47	25	46	0
HRV14 and RND HRV14	43	45	25	36	0
HRV-C and RND HRV-C	44	45	24	37	0
HRV-A and RND HRV-A	43	45	24	36	0
HRV2 and RND HRV2	42	43	23	37	0

### Comparison of the tCP-profiles of the serotypes of HRV and FMDV polyprotein coding ORFs

**Figure 2:**
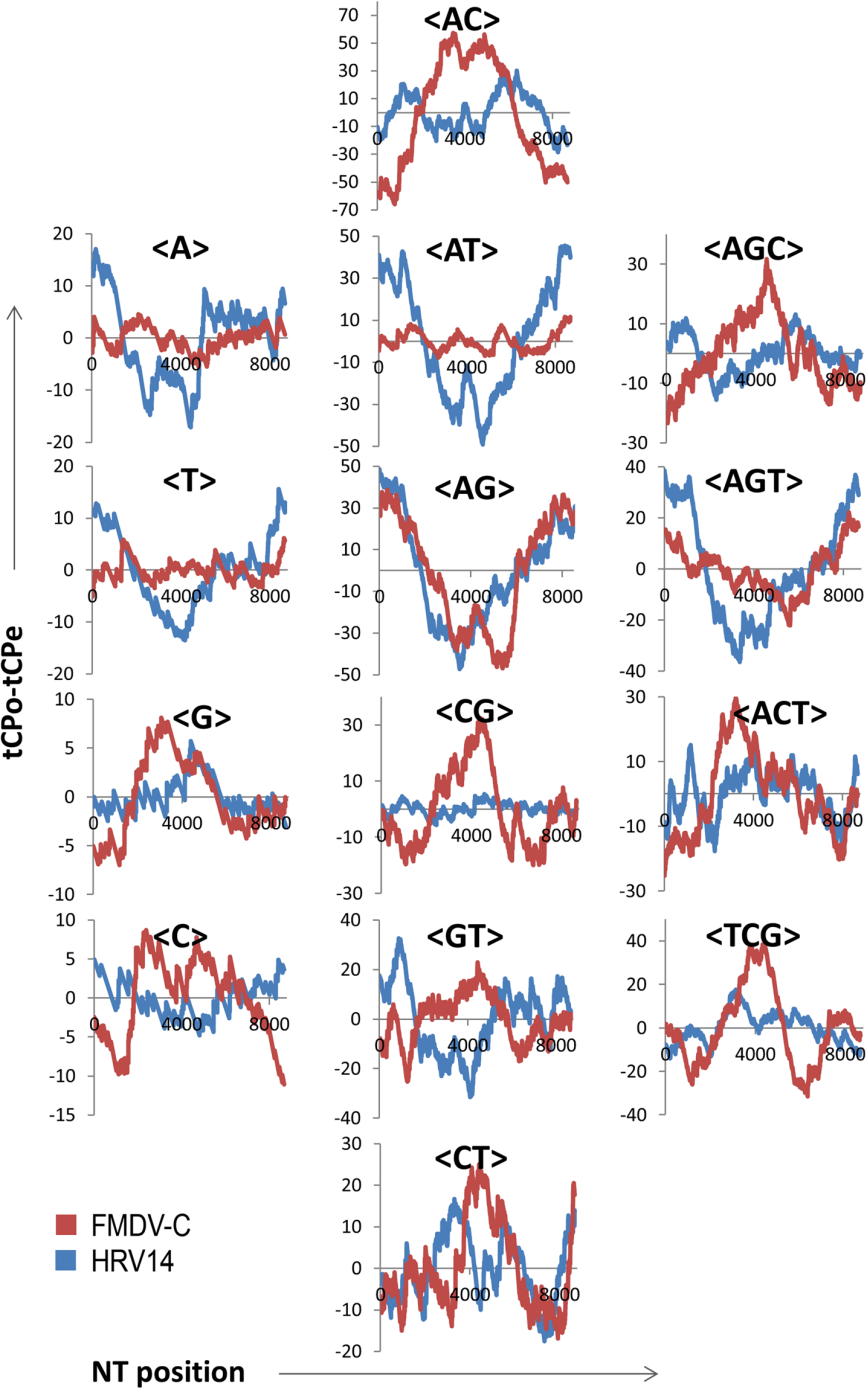
Comparison of the 14 tCP-profiles obtained from the alignments of the tCP-sequences of FMDV-C (red) and HRV14 (blue) polyproteins.

Figure 2 shows the panel of 14 tCP-profiles resulting from the alignment of the tCP-sequences of the polyprotein coding ORFs of two serotypes of HRV and FMDV (HRV14 and FMDV-C). The tCP-profile of <AG> is the only one showing a significant resemblance along the RNA sequences of both species. In fact, the profiles of the tCP <AG> between HRV14 and FMDV-C shows a high correlation coefficient (r = 0.86) when compared with those observed in the remaining 13 tCP-profiles, all of them showing notably lower correlation coefficients. The analysis of the distribution of the tCP <AG> along the length of HRV14 and FMDV-C polyprotein coding ORFs (Fig. 2, panel <AG>) reveals three compositional regions clearly differentiated in the alignment of the tCP-sequences of HRV14 and FMDV-C, responsible of the high correlation coefficient observed for the tCP <AG>. The regions 5′ and the 3′ which extend in the alignment around ¼ of the whole show in both picornaviruses positive differences relative to the observed and the predicted <AG>events. Such differences are negative in the central region with an extension in the alignment of around ½ of the whole. Thus, the patterns of decrease and increase in the <AG>-usage along the length of HRV14 and FMDV-C polyprotein coding ORFs shows the presence of a common pattern in the use of the nearest neighbour triplets formed by the NTs A and G shared by both genomes. In consequence, both ORFs sharing the tCP <AG> and the associated NT pattern suggest the existence of a possible scaffold-like compositional structure interspersed in the polyprotein coding ORFs of both viruses.

A similar analysis with the other serotypes of FMDV and HRV was carried out and the results are shown in Table 3. The table displays the conserved tCPs and the correlation coefficients of all sequences analyzed. The number of conserved TCPs shared by FMDV serotypes was significantly higher than the number of conserved TCPs shared by HRV serotypes. The correlation coefficients observed were also noticeably higher when the serotypes of FMDV were compared than when the serotypes of HRV were compared. In many cases, the tCP-profiles with correlation coefficients <0.85 show coincidences in short fragments of tCP-sequences of FMDV and also of HRV. There are not many shared tCPs between serotypes of HRV and FMDV. There are 16 possible alignments between the polyprotein-coding ORFs of HRV and FMDV serotypes. The tCP <AG> is shared in 2 alignments out of 16 (FMDV-A vs HRV14 and FMDV-C vs HRV14) and the tCP <AC> in one out of 16 (FMDV-SAT1 vs HRV-A). The remaining alignments do not show coincidences higher than the cut-off (Table 3). However, it can be observed some profiles (Table 3, tCPs underlined) with correlation lower but near the cut-off mainly of the tCPs <AG> and <AC>.

**Table 3:** Conserved tCPs and correlation coefficients (r) between serotypes of FMDV and HRV.

Serotype	FMDV-A	FMDV-C	r**	FMDV-SAT1	r	FMDV-O	r	HRV14	r	HRV-C	r	HRV-A	r	HRV2	r
FMDV-A	All tCPs^*^	<A>	0.85	<AC>	0.94	<A>	0.87	<AG>	0.85	—	—	—	—	—	—
		<AC>	0.94	<AG>	0.98	<AC>	0.88	<AGT>	0.83						
		<AG>	0.98	<AGT>	0.91	<AG>	0.99								
		<AGT>	0.97	<TCG>	0.86	<CT>	0.86								
		<ACT>	0.86			<AGT>	0.95								
FMDV-C		All tCPs		<G>	0.89	<C>	0.91	<AG>	0.86	—	—	<AC>	0.81	—	—
				<AC>	0.95	<AC>	0.95								
				<AG>	0.99	<AG>	0.99								
				<CT>	0.86	<CT>	0.87								
				<AGT>	0.91	<AGT>	0.93								
						<TCG>	0.88								
FMDV-SAT1				All tCPs		<AC>	0.85	<AC>	0.82	<AG>	0.81	<AC>	0.85	<AG>	0.80
						<AG>	0.98								
						<AGT>	0.91								
FMDV-O						All tCPs		—	—	—	—	—	—	—	—
HRV14								All tCPs		<A>	0.86	<TCG>	0.87	<AG>	0.89
										<AG>	0.98				
										<AC>	0.91				
										<AT>	0.86				
HRV-C										All tCPs		<G>	0.87	<AG>	0.85
												<AC>	0.90		
												<AT>	0.91		
HRV-A												All tCPs		<AT>	0.85
														<AG>	0.87
HRV2														All tCPs	

^*^All tCPs indicate that when compared two identical tCP (or NT) sequences all tCPs (or all NTs) are conserved. Underlined; correlation coefficients lower but near the cut-off.

### tCP-sequence alignments of the serotypes of FMDV polyprotein coding ORFs and their randomized counterparts

**Figure 3:**
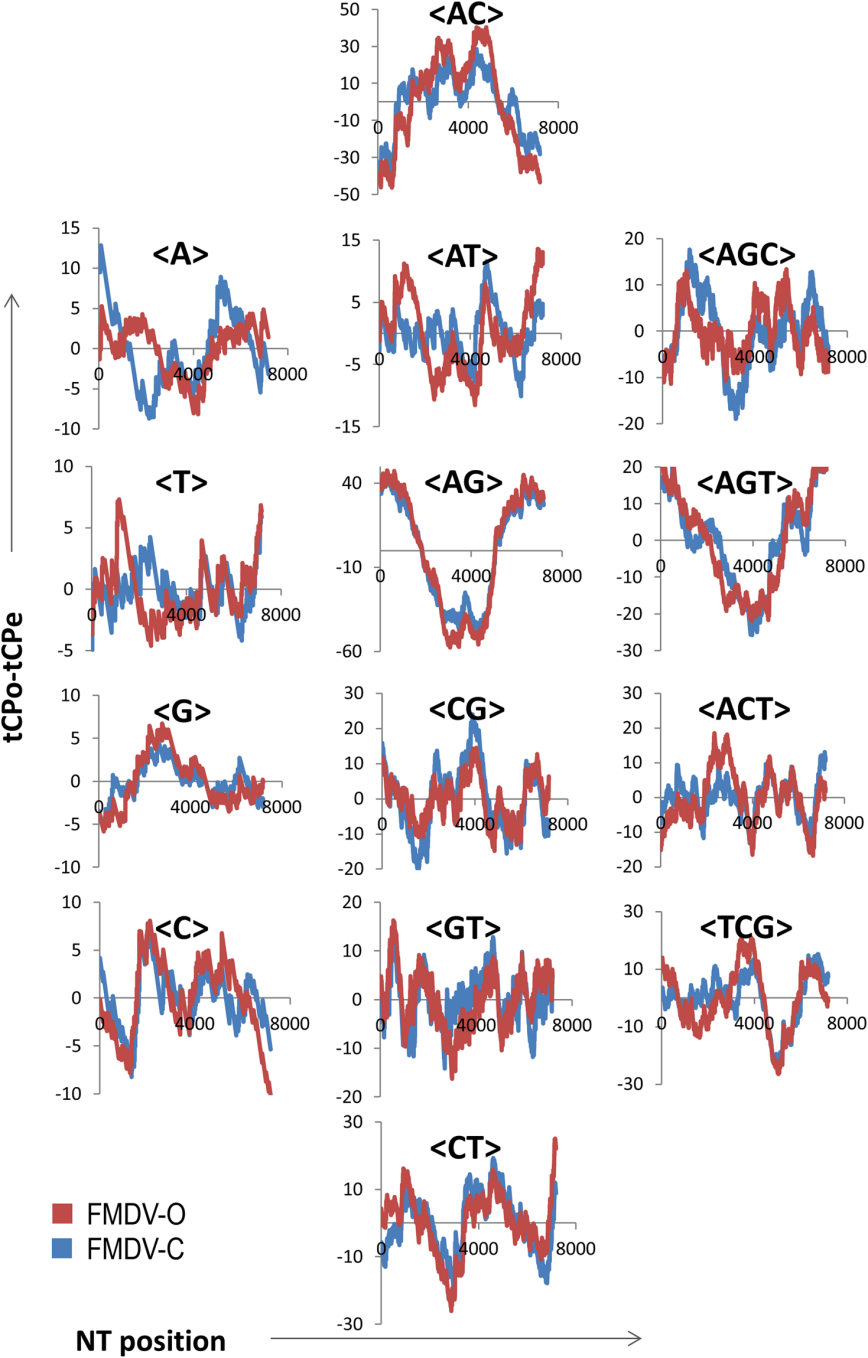
Comparison of the 14 tCP-profiles obtained from the alignments of the tCP-sequences of FMDV-C (blue) and FMDV-O (red) polyproteins.

To prove the existence of a possible scaffold-like compositional structure shared by the polyprotein coding ORFs of HRV and FMDV it is required to demonstrate first that such structures are also present in other different serotypes of both species. To show whether the tCP-profiles of <AG> and <AC> are also common in other divergent serotypes of FMDV we have included as an example a panel (Fig. 3) comparing the 14 tCP-profiles of two serotypes of FMDV (FMDV-C and FMDV-O). The tCP-profiles show a high level of resemblance for the tCPs <G>, <C>, <AG>, <CG>, <AC> and <AGT>. We observed correlation coefficients ranging from r = 0.86 for <C> to r = 0.99 for <AG>. It was also observed that the polyprotein-coding ORFs of FMDV-C and FMDV-O share six-fold degenerate tCPs (<AG>, <CG>, <AC> and <AGT>) and two non-degenerate tCPs (<G> and <C>) (see Table 1). As can be noted the tCPs <AG> and <AC> are shared by both serotypes of FMDV with the highest correlation coefficient. A similar analysis was carried out for all FMDV serotypes (FMDV-A, FMDV-C, FMDV-SAT1 and FMDV-O) and the results are summarized in Table 3. I was observed that the tCP <AG> was present in all FMDV serotypes analyzed with the highest correlation coefficients (r > 0.98 in all cases) and the tCP <AC> was also present in all FMDV serotypes with high correlation coefficients (r > 0.94) with two exceptions (r > 0.85). The tCPs <AG> and <AC> were not the only shared tCPs in FMDV. In fact, all serotypes of FMDV analyzed share also the tCP <TCG> with high correlation coefficients (r ≥ 0.91) as an indication that <AG>, <AC> and <TCG> are genus specific. Other tCPs conserved in some FMDV serotypes were observed (such as <A>, <G>, <C>, <ACT>, <TCG> or <CT>; Table 3) although they were not common to all FMDV serotypes.

We also compared the tCP-sequence of FMDV serotypes with their randomized sequence counterparts to discard the possibility of random fits. We have shown as an example in Supplementary Fig. 1 the tCP panel representing the 14 tCPs of the alignment of FMDV-C relative to their randomized counterpart. The panels for FMDV-A, FMDV-SAT1 and FMDV-O gave similar results. No significant correlation was found when comparing the FMDV serotype sequences with the average of its randomized counterparts (Table 2). The highest correlation coefficient observed was r = 0.46. This result suggests that the polyprotein coding ORF of the FMDV serotypes is far from random despite of the data shown in Table 2. Finally, as an example, we compared the profile of the FMDV-C polyprotein coding ORF with itself to test the reliability of the software obtaining the value of r = 1, as expected.

### tCP-sequence alignments of the serotypes of HRV polyprotein coding ORFs and their randomized counterparts

**Figure 4:**
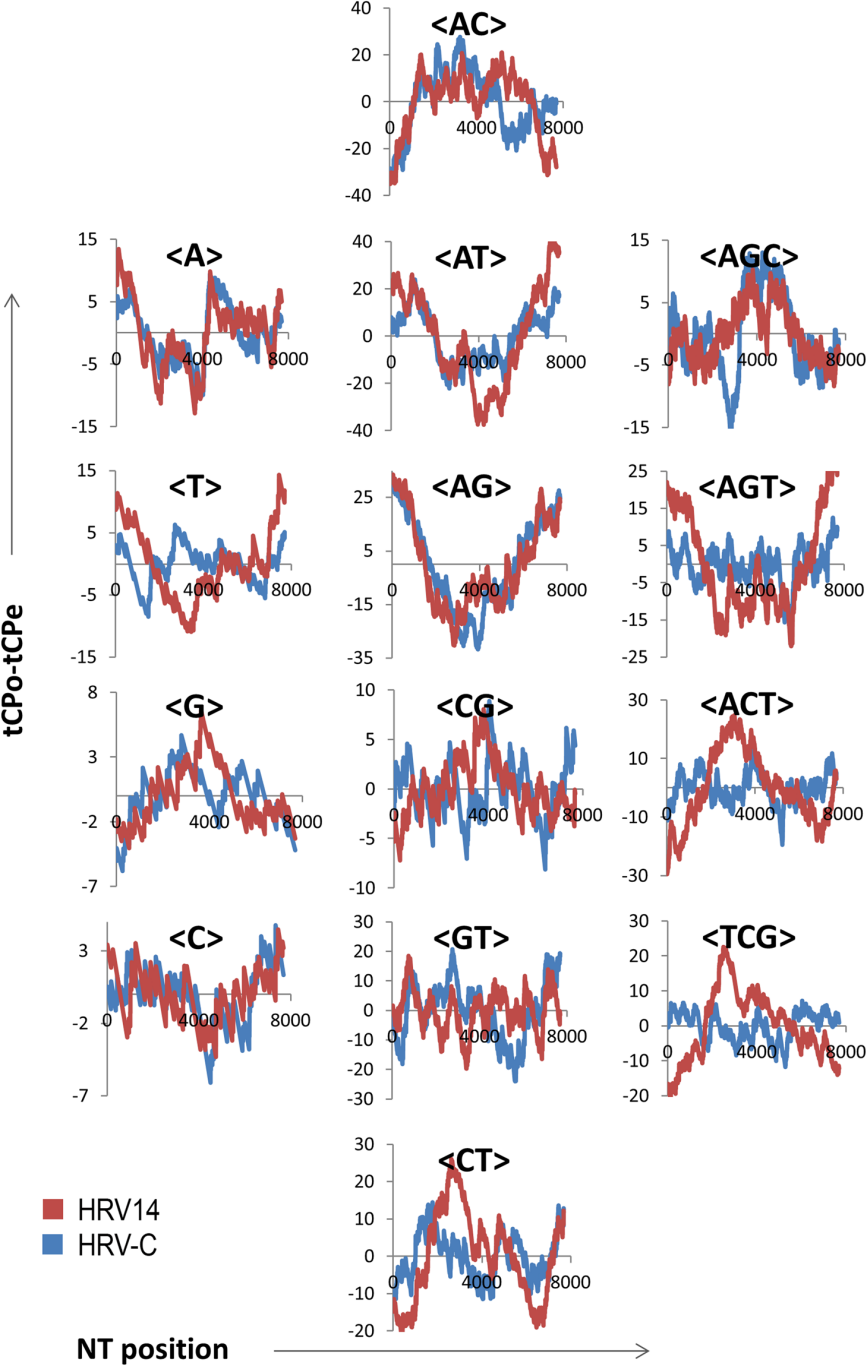
Comparison of the 14 tCP-profiles obtained from the alignments of the tCP-sequences of HRV14 (red) and HRV-C (blue) polyproteins.

In order to show whether the profiles <AG> and <AC> are also common in the different HRV serotypes analyzed, Fig. 4 shows, as an example, the panel comparing the 14 tCP-profiles resulting from the alignment of the tCP-sequences of the HRV serotypes HRV14 and HRV-C. As can be observed, the tCP-profiles of HRV14 and HRV-C show a high level of resemblance for the tCPs <A>, <AC>, <AG> and <AT>. Thus, in these serotypes of HRV the tCPs <AG> and <AC> are present with high correlation coefficients. Thus, HRV14 and HRV-C polyprotein coding ORFs share three-fold degenerate (<AC>, <AG> and <AT>) and a non-degenerate (<A>) tCPs (see Table 1).

When the remaining serotypes of HRV (HRV14, HRV-C, HRV-A and HRV2) were compared (Table 3), the tCP <AG> was also present in the majority of the alignments, with high correlation coefficients (0.85 ≤  r ≤  0.90). Some of the HRV serotypes shared also the tCPs <AC> and <AT> with high correlation coefficients (0.86 ≤ r ≤ 0.90). Other tCPs (such as <A>, <G>, <TCG>, <ACT>, <TCG>; Table 3) were conserved in some HRV serotypes although they were not common to all HRV serotypes. However, when we compared the HRV14, HRV-C, HRV-A and HRV2 polyprotein coding ORFs and its randomized counterparts we did not observe any conserved tCP (the highest correlation coefficient observed was r = 0.49). We have represented, as an example in Supplementary Fig. 2, the tCP panel representing the 14 tCPs of the alignment of HRV14 relative to their randomized sequence counterpart. The panels of HRV-C, HRV-A and HRV2 gave similar results (Table 2). These results suggest that the serotypes of HRV14 polyprotein-coding ORF analyzed are far from random and that the serotypes of HRV share the tCP <AG> (Table 3). As expected, the correlation coefficient between the HRV14 polyprotein-coding ORF with itself is r = 1.

In summary, the comparison of HRV and FMDV serotypes shows that despite the low sequence similarity of the polyprotein coding ORFs of these highly divergent picornaviruses some serotypes share common tCPs as <AG> and <AC>(Table 3) generating interspersed compositional structures common in some serotypes of both species.

### Is the tCP scaffold-like compositional structure under selection constraints?

**Figure 5:**
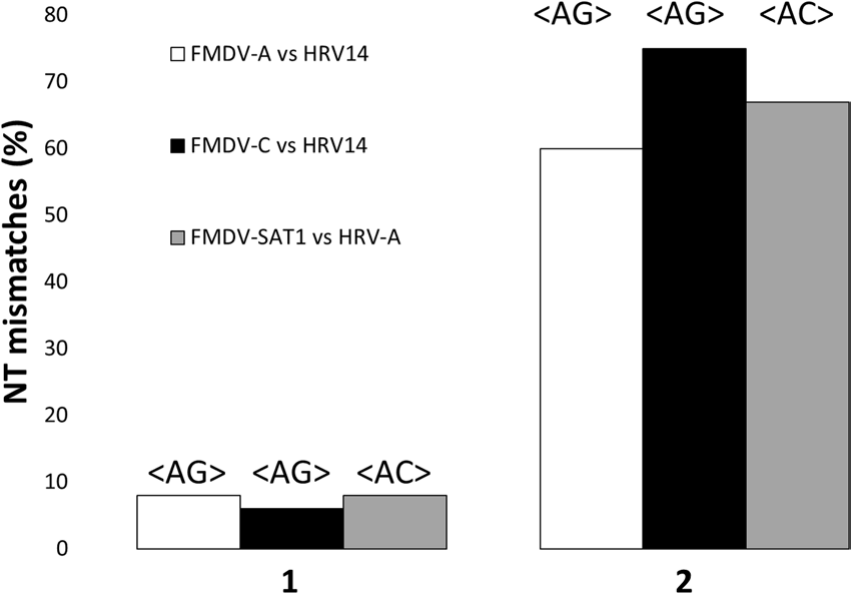
Bar diagram showing the percent of NT-mismatches in genomic regions in which FMDV-A and HRV14 (white bars) share the tCP <AG> (1) relative to the percent of NT-mismatches observed in genomic regions in which only one of the species has the tCP <AG> (2). The figure also represents the mismatches of the tCP <AG> shared by FMDV-C and HRV14 (black bars) and the mismatches of the tCP <AC> shared by FMDV-SAT1 and HRV-A (grey bars).

We analyzed whether the <AG> emergent compositional structure common to HRV14, FMDV-C and FMDV-A is under selection constrain for variability. In order to show that those hypothetical scaffold-like structures are under selection constraint, we analysed the NT-mismatches in the genomic regions in which both, HRV14 and FMDV-C on one hand and HRV14 and FMDV-A on the other, share the tCP <AG>, and also in the regions in which only one of the virus species contain the tCP <AG>. Figure 5 shows the percent of NT-mismatches in genomic regions in which the HRV or FMDV serotypes analyzed share the tCP <AG> or <AC> (bar 1) relative to the percent of NT-mismatches observed in genomic regions in which only one of the virus species has the tCP <AG> or <AC> (bar 2). The genomic regions in which HRV14 and FMDV-C share the tCP <AG> have 42 mismatches out of 519 NTs (8%), while the regions in which only one of the species has <AG> have 1089 mismatches out of 1817 NTs (60%). Similar results were obtained for HRV14 and FMDV-A for the shared tCP <AG>. In this case the genomic regions in which both HRV14 and FMDV-A share the tCP <AG> have 39 mismatches out of 613 NTs (6%), while the regions in which only one of the species has <AG> have 1208 mismatches out of 1622 NTs (75%). Finally, in the case HRV-A and FMDV-SAT1, the genomic regions in which both HRV-A and FMDV-SAT1 share the tCP <AC> have 62 mismatches out of 764 NTs (8%), while the regions in which only one of the species has <AC> have 1360 mismatches out of 2029 NTs (67%). These data suggest that the shared <AG> regions between i) HRV14 and FMDV-C and ii) HRV14 and FMDV-A are under evolutionary constraints for variability and also the shared <AC>regions of HRV-A and FMDV-SAT1.

All these data together with those illustrating the low degree of NT-similarity (similar to random) between the RNAs of (i) HRV14 and FMDV-C, (ii) HRV14 and FMDV-A and (iii) HRV-A and FMDV-SAT1 polyprotein coding ORFs (Table 2) reinforce the idea of the <AG> and <AC>conservation in some genomes of the *Picornaviridae* family, and consequently of its associated NT-compositional structures. The predominance in the conservation of the tCPs <AG> and <AC> is supported by data indicating that the bulk of tCPs with correlation coefficients lower but near the cut-off are <AG> or <AC> (see data underlined in Table 3). The results presented support the hypothesis that despite the low similarities found in the NT and tCP-sequences of the polyprotein coding ORFs of HRV14, FMDV-A and FMDV-C and also of HRV-A and FMDV-SAT1, they share common interspersed compositional structures that are highly conserved and subjected to strong constraints during evolution (Tables 2 and 3). The high correlation coefficients between the polyprotein coding ORFs of (i) HRV14 and FMDV-A, (ii) HRV14 and FMDV-C for the tCPs <AG> and (iii) HRV-A and FMDV-SAT1 for the tCP <AC> and the low correlation shown by the remaining tCPs indicate that the tCPs <AG> and <AC>, and their associated NT-stretches could be important for the fitness of (i) HRV14 and FMDV-C, (ii) HRV14 and FMDV-A, and (iii) HRV-A and FMDV-SAT1, since they are conserved along the speciation events of both species.

### The tCP-profile and the genome organization of FMDV and HRV

**Figure 6:**
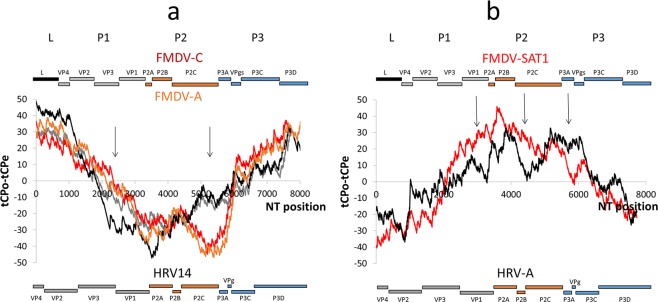
Differences between tCP-observed (tCPo), and estimated (tCPe) events for the conserved tCPs in the alignment of the polyprotein coding ORFs of FMDV and HRV. An schematic of the RNA genomic organization of the polyprotein-encoding ORFs of FMDV and HRV are superimposed to the tCP-profiles according to the L-P1-P2-P3 structural scheme^24^. (**a**) The L, P1, P2 and P3 genomic organization of FMDV-A, FMDV-C and HRV14 are in black, grey, orange and blue colours. L represents in FMDV-A and FMDV-C, the leader proteinase; HRV14 lacks this region in the polyprotein coding ORF. P1 contains the sequences coding for viral structural proteins: VP4, VP2, VP3 and VP1. P2 contains the sequences coding for non-structural genes 2 A, 2B, and 2 C. P3 contains the sequences coding for non-structural genes 3 A, 3B, 3 C, and 3D. The tCP-profiles are represented as follows: FMDV-A (red), FMDV-C (orange) and HRV14 (in black, the alignment FMDV-C vs HRV14 and in grey, the alignment FMDV-A vs HRV14). Arrows underline significant differences between tCPo and tCPe events between FMDV-A, FMDV-C and HRV14 polyprotein-encoding ORFs. (**b**) The genomic organization of FMDV-SAT1 and HRV-A are as indicated before. The tCP-profiles are represented as follows: FMDV-SAT1 (red) and HRV-A (black).

Figure 6 shows the shared similarities and dissimilarities, at a local level, between the <AG> and <AC>-usages observed (tCPo) and the estimated (tCPe) in the alignments of (i) FMDV-C and HRV14, (ii) FMDV-A and HRV14 and (iii) FMDV-SAT1 and HRV-A polyprotein coding ORFs relative to their genome organization. Superimposed to the <AG> profile, an schematic representation of the RNA genomic organization of FMDV and HRV polyprotein coding ORFs may be observed according to the known L-P1-P2-P3 structural scheme^24^. The FMDV leader proteinase (named L) is an additional N-terminal protein present in some picornavirus genera that can either be a papain-like cysteine proteinase or have another function depending on the virus genus^25^. P1 represents the region of viral genes coding for structural proteins and P2 and P3 represent the regions coding for non-structural proteins.

Analysis of the distribution of the conserved tCP <AG> along the polyprotein coding ORFs of HRV14, FMDV-C or FMDV-A revealed 3 regions that can be clearly differentiated regarding <AG>-usage (Fig. 6a). These regions are responsible for the high correlation coefficient observed between the <AG>-profiles of the viral genomes. The 5′ and 3′ regions of the polyprotein-encoding ORFs are characterized by <AG>-usages higher than expected in both species, whereas in the central region <AG>-usages are lower than expected. The L region in FMDV-C and FMDV-A is characterized by a constant excess of the <AG>-usage similar to that observed in the VP4 region and in the 5′ region of VP2 in HRV14. The similar <AG>-usages observed in those regions suggest that the L region in FMDV-C and FMDV-A and the VP4 region in HRV14 are characteristic of genera, creating a scaffold-like compositional structure shared by both picornaviruses in spite of the deep functional divergence observed in those RNA fragments. The P1 region, coding for the structural proteins VP4, VP2, VP3 and VP1 is characterized by a continuous decrease in the <AG>-usage in HRV14 except in the L region of FMDV-C. The decrease in <AG>-usage is more pronounced in HRV14 than in FMDV-C but not in HRV14 than in FMDV-A (see arrow in the P1 region in Fig. 6a) showing, therefore, a significant difference in the <AG>-distribution between i) HRV14 and FMDV-A, and ii) HRV14 and FMDV-C. The same occurs in the P3 region of both HRV14 and FMDV-C polyprotein coding ORFs where <AG>-usages are higher than expected. The P3 region contains the sequences coding for the non-structural proteins P3A, P3B (or VPg), P3C and P3D of HRV14 and FMDV-C. The more important difference observed at local level between HRV14 and FMDV-C is in the P2 region. The minimum <AG>-usage is observed in the central part of the P2C protein coding region of FMDV-C while this is not observed in the equivalent region of HRV14.

Figure 6b shows the distribution of the conserved tCP <AC> along the HRV-A and FMDV-SAT1 polyprotein coding ORFs. The rest of the tCPs do not correlate at all. The figure shows two regions that can be clearly differentiated according to <AC>-usage and that are responsible for the high correlation coefficient observed between the <AC>-profiles in both viral genomes. A region is characterized by a continuous increase in <AC>-usage and the other region by a continuous decrease in <AC>-usage. The maximum of <AC>-usage was found near the limit between the P1 and P2 regions of the FMDV-SAT1 and HRV-A genomes. Some variability was observed in some regions of both polyprotein-encoding ORFs (see arrows) such as the VP1, P2C and also P3A and VPg regions, suggesting that these regions could have diverged during speciation events.

The data indicate that the L and P3 regions in FMDV-C and FMDV-A, and VP4 and P3 regions in HRV14, are evolutionarily more conserved than the P1 and P2 regions exhibiting similar usages of the conserved tCP <AG>. Therefore, those regions may be subjected to stronger evolutionary constraints for variability than the structural protein region P1 and the non-structural protein region P2. The data shown are partly supported by studies on the variability of the polyprotein region of the FMDV genome^26^. Regarding FMDV-SAT1 and HRV-A, the data suggest that the similar distribution of <AC> along their genomes may be important for the biological fitness and survival of both viruses. As a conclusion, the conservation of <AG>in (i) HRV14 and FMDV-C and (ii) HRV14 and FMDV-A and the conservation of <AC> in FMDV-SAT1 and HRV-A may provide a signature to locate conserved fragments in those genomes with likely biological relevance.

## Discussion

The method based on the tCP concept^10^ is useful to look for similarities and differences of genomic sequences and it has been employed as a gene clustering system^10,11^, to study the co-evolution of introns and exons in human-mouse orthologs^12^ and also for the study of homology of a high number of genes coding for an important tCP-cluster of transmembrane proteins^11^. In this paper we use a variant of the tCP-method^13^ to study the possible existence of a common gene structure shared by two highly divergent picornaviruses having a very low RNA sequence identity, which may have some evolutionary implications. The main difference between a system based on NT-content and other based the tCP-content have to do with the fact that the NT-content approach counts how many NTs of different types are contained in the sequence, whereas the tCP-content approach counts the nearest-neighbours of each NT in the sequence, taking into account, thus, the context of each nucleotide and categorizing all nearest neighbours according to their gross composition (Table 1). This method differs from others in that the sequence is read in fully overlapping way and, thus, independently of the reading frame. We would like to stress that reading in that way two-thirds of the triplets that are extracted from the sequence are not codons (they do not code for any amino acid). These extra triplets are responsible for one source of genome variability that is directly related with the synonymous codon-usage.

HRV and FMDV are characterized by similar genomic distributions in both polyprotein coding ORFs^16^ among other functional features, despite the high divergence in their genomes and the low sequence identity observed between them^26–28^. The dissimilarity observed is very similar to random at NT and tCP levels (Table 2). However, the data show that the HRV and FMDV polyprotein-encoding ORFs share, in some of their serotypes, the tCP <AG> as occur in (i) FMDV-A and HRV14 and (ii) FMDV-C and HRV14, and the tCP <AC> in HRV-SAT1 and HRV-A. This underlying similarity between some serotypes of HRV and FMDV could be useful to predict critical mutation points and to supply new sequence motifs important from the evolutionary point of view.

On the other hand, it has been reported^16^ that in picornaviruses, the discordance in tree topology among datasets highlights divergences in evolutionary parameters such as rates of evolution, selection pressures and possible past recombination events. Data indicating that changes in phenotype are not closely correlated with speciation support the idea that viral phenotypes respond rapidly to selection. Consequently, the picornaviruses would exploit many adaptive solutions not associated with their evolutionary history^16^. However, in spite of the fact that HRV14 and FMDV-C display a broad range of genomic variation and phenotypic flexibility making difficult their classification and identification^7,8^, the data obtained in this paper show that both picornaviruses maintain and share subtle common scaffold-like compositional structures (Fig. 2 and Table 3). This fact could restore the possibility of past recombination events on picornavirus and also the existence of a close link between changes in phenotype and speciation facilitating their classification and identification.

The controls described in this paper have been designed mainly with two objectives: (i) to demonstrate that the tCP-usage of HRV and FMDV polyprotein coding ORFs are far from those of their randomized genomes despite HRV and FMDV have percent identities close to random and (ii) to support the existence of new scaffold-like compositional structures that are shared by some of the serotypes analyzed. The results obtained from the analysis of the controls demonstrate the absence of random fits when HRV and FMDV polyprotein coding ORFs were compared with their randomized sequences. The average of 5 randomized genomes for HRV and FMDV assures that random fits do not exist between them. This result guarantees the coherence of the panel when the tCP-sequences of HRV and FMDV are compared.

The method proposed predicts the existence of new scaffold-like compositional structures shared by (i) HRV14 and FMDV-C and (ii) HRV14 and FMDV-A and also by HRV-A and FMDV-SAT1. The results of this analysis are consistent with results previously obtained by other methods. We observed for example that FMDV-C and FMDV-O share 6 tCPs whereas HRV14 and HRV-C share only 4 tCPs, which is consistent with the results of a classic sequence analysis regarding the evolutionary divergence of FMDVs relative to that of HRVs^22,23^. The same can be observed in Table 3 for all FMDV serotypes analyzed. These data indicate, as expected, that FMDV serotypes have diverged from each other less than the HRV serotypes. Something similar occurs when the HRV serotypes were compared with those of FMDV. The data suggest the possibility to do phylogenetic analysis with tCP sequences.

Relative to the close connection between the shared tCPs <AG> and the genome organization of FMDV-C, FMDV-A and HRV14 it was observed that the high <AG>-usage detected in the L region that codes for the FMDV L proteinase suggests that this region is important for fitness and survival of FMDV. In fact, the L protein has been described as an important determinant of virulence^29^ that despite of slightly affecting the replication rate^30^ exerts a low ability to cause lesions^31^ and shut off the host cap-dependent mRNA translation allowing the virus to use the host cell protein synthesis machinery^32–34^. In HRV14, which lacks the L region, we observed <AG>-usages higher than expected affecting stretches coding for the structural proteins VP4 and VP2 as an indication that these proteins are more conserved than the other structural proteins showing <AG>-usages lower than expected, as is the case of VP3 and VP1. The data could explain why VP4 and VP2 have been described as a more stable cleavage intermediate, called VP0 that may perform functions other than those of their individual constituents^29^.

Relative to the non-structural proteins of regions P2 and P3 of FMDV-C, FMDV-A and HRV14 polyprotein-encoding ORFs we observe that the P2 region displays the most important difference regarding <AG>-usage, lower than expected in FMDV-C and FMDV-A and absent in HRV14. The data suggest that a minimum <AG>-usage (Fig. 6a) is characteristic of genera because it is present in FMDV serotypes FMDV-C, FMDV-A, FMDV-SAT1 and FMDV-O but absent in the HRV serotypes HRV14, HRV-A, HRV2 and HRV-C (Figs 3 and 4). The minimum <AG>-usage in FMDV occurs within the region coding for protein 2C. The data in Fig. 6a support the fact that this protein highly conserved among the viral proteins encoded by FMDV has been described as responsible for many biological functions linked to membrane targeting^29^. The high conservation of this protein in FMDV and the common <AG>-profile (Fig. 6a) suggest that 2C could be a protein specific of genera. The data referred to FMDV-SAT1 and HRV-A (Fig. 6b) indicate that the P2 region also displays significant differences regarding <AC>-usage, higher than expected in FMDV-SAT1 relative to HRV-A. As already mentioned protein 2C is highly conserved relative to other FMDV proteins^29^. The differences observed among FMDV-SAT1 and HTV-A also support the hypothesis that 2C would be a protein specific of genera. As occur with FMDV-A, FMDV-C and HRV14, the P3 region of FMDV-SAT1 and HRV-A are also characterized by different <AG>-usages suggesting that all of them have diverged during speciation events.

This comment could also be extended to the structural protein VP3 and to the non-structural protein 3C (see arrows in Fig. 6a). On the other hand, in the P3 region the <AG>-usage is higher than expected, especially in the region coding for the virus-encoded RNA-dependent RNA polymerase that plays an important role in the life cycle of RNA viruses^29^. There are data indicating that the differences in <AG>-usage between FMDV-C and HRV14 polyprotein coding ORFs (see arrows) disappear when closely related serotypes of FMDV as FMDV-C, FMDV-A, FMDV-SAT1 and FMDV and HRV as HRV14, HRV-A, HRV2 and HRV-C (Figs 3 and 4, tCP-profile <AG>) are compared as an indication that such differences may appear during HRV and FMDV speciation events.

Due to the nature of the tCP definition (see Table 1) the common tCP conserved motifs may contain NT mismatches^13^. Thus, to search for common motifs a convincing model should be proposed. Simple models are designed to search for conserved motifs of fixed length. However, more advanced models would incorporate variability like insertions and deletions. A shortcoming of the actual models is the lacking of background sequences in which a motif is hidden^35^. The tCP method supply a variety of new common motifs interspersed in HRV and FMDV genomes.

## Supplementary information

Supplementary Information

## Data Availability

Materials, data and associated protocols are promptly available to readers without undue qualifications in material transfer agreements.
