# Supplementary material for: Evolutionary conserved compositional structures hidden in genomes of the foot-and-mouth disease virus and of the human rhinovirus
Source: Sci Rep. 2019 Nov 12;9:16553. doi: 10.1038/s41598-019-53013-8 (PMC6851159; doi:10.1038/s41598-019-53013-8)

Evolutionary conserved compositional structures hidden in genomes of the foot-and-mouth disease virus and of the human rhinovirus

Miguel Angel Fuertes*, Silvia López-Arguello, Carlos Alonso

Supplementary Figure 1. tCP-sequence comparison between the FMDV-C polyprotein coding ORF with the average of 5 randomized sequences generated by the application shuffleseq (EMBOSS) to the FMDV-C polyprotein coding ORF.

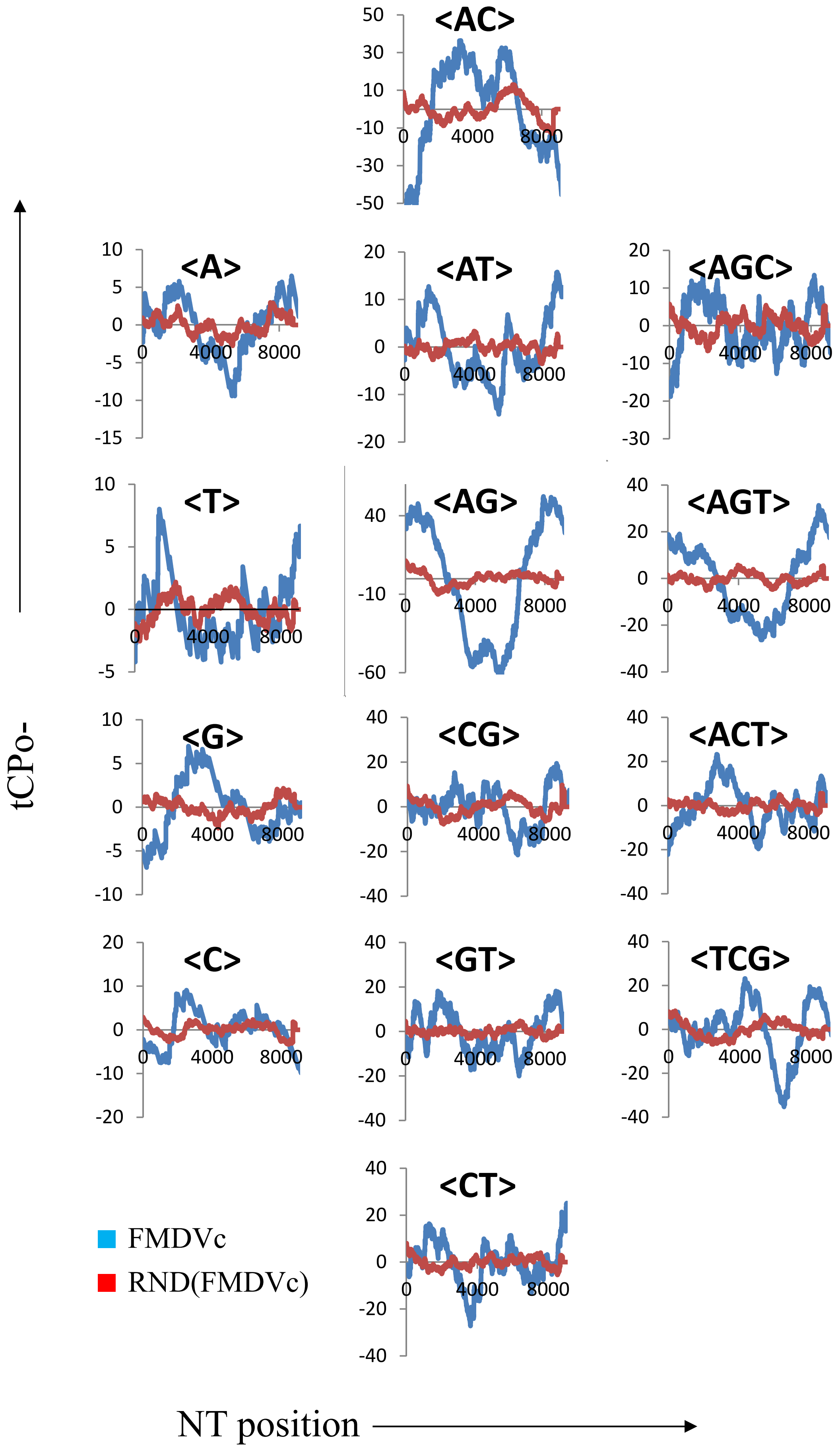

Supplementary Figure 2. tCP-sequence comparison between the HRV14 polyprotein coding ORF with the average of 5 randomized sequences generated by the application shuffleseq (EMBOSS) to the FMDV-C polyprotein coding ORF.

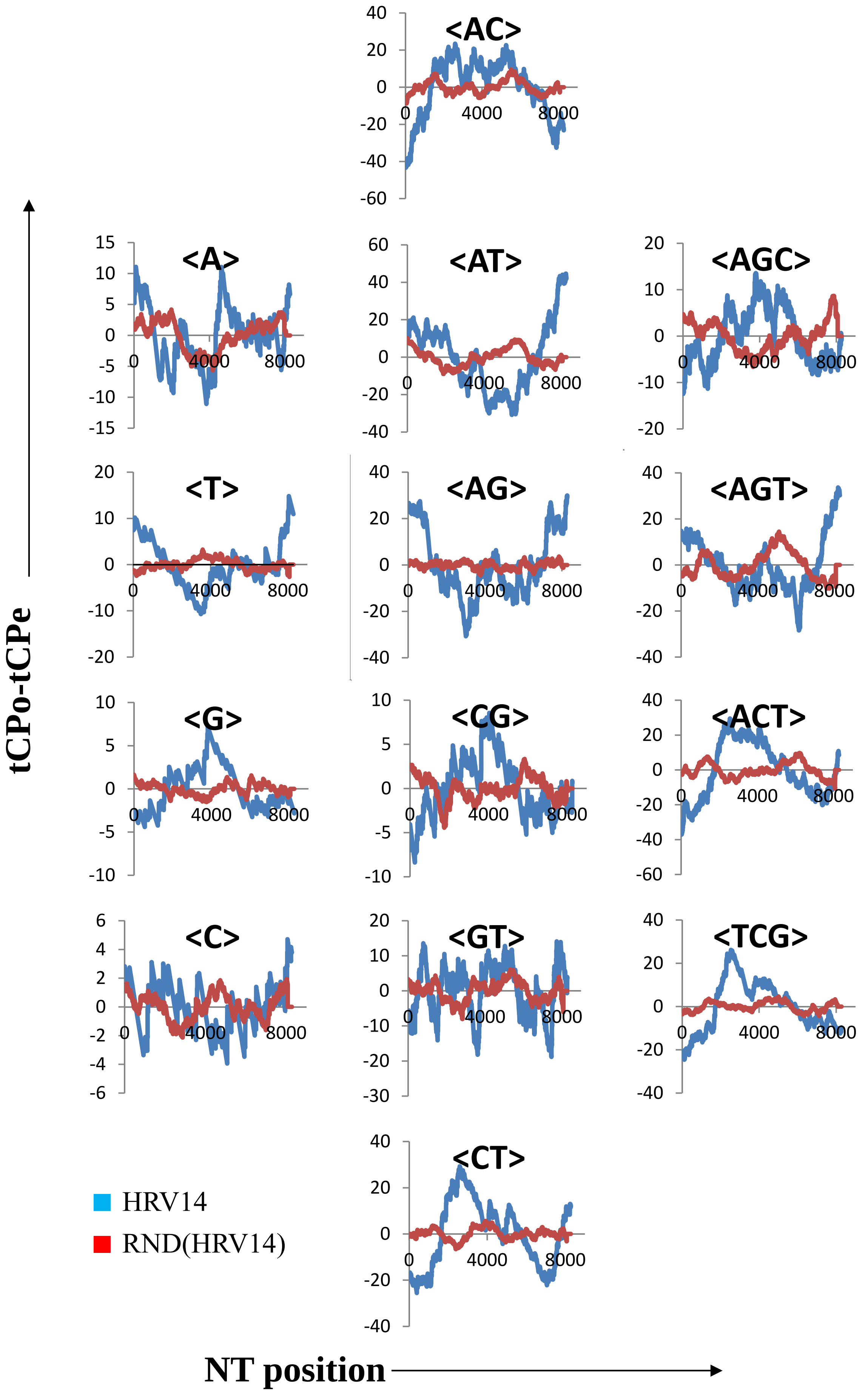

Supplement: Supplementary file 1 — Supplementary Information [file 41598_2019_53013_MOESM1_ESM.pdf]
